# Case Report: A novel variant of the TTN gene and two other rare variants in a Chinese patient with dilated cardiomyopathy

**DOI:** 10.3389/fcvm.2025.1527544

**Published:** 2025-04-09

**Authors:** Shan Han, Ying-Yi Zhang, Jie Geng

**Affiliations:** Department of Cardiology, Chest Hospital, Tianjin Key Laboratory of Cardiovascular Emergency and Critical Care, Tianjin Municipal Science and Technology Bureau, Tianjin University, Tianjin, China

**Keywords:** TTN gene variant, dilated cardiomyopathy, rare variants, left ventricular reverse remodeling, case report

## Abstract

Genetic factors are estimated to cause approximately 30%–50% of dilated cardiomyopathy (DCM) cases, with Titin (TTN) being the most commonly implicated gene, accounting for 20%–25% of genetic causes. Many DCM-causing TTN mutations are heterozygous truncating variants, including frameshift, non-sense, and essential splice site mutations. SCN5A mutations are associated with arrhythmias, while pathogenic variants in the low-density lipoprotein receptor (LDLR) gene are associated with familial hypercholesterolemia. Here, we report a case of DCM with a novel TTN variant, as well as two rare variants in the SCN5A and LDLR genes. It is rare for a patient to have three rare genetic variations and this may expand the genetic map of DCM and TTN, offering important insights for future studies on their genetic and disease relationships.

## 1 Introduction

Dilated cardiomyopathy (DCM) is a cardiac disorder characterized by left ventricular (LV) dilatation and systolic dysfunction, occurring in the absence of known abnormal loading conditions or significant coronary artery disease (1). Currently, over 50 genes encoding cytoskeletal, nuclear skeleton, mitochondrial, and calcium-handling proteins have been linked to hereditary DCM. Titin (TTN) is the most frequently implicated gene, accounting for 20%–25% of genetic causes, followed by lamin A/C (LMNA) at approximately 6% and beta-myosin heavy chain (MYH7) (2). Although SCN5A mutations are primarily associated with arrhythmias, they can also cause DCM (3). The pathogenic variants in the low-density lipoprotein receptor (LDLR) gene have been known to cause familial hypercholesterolemia (FH).

Here, we describe a case of DCM with a novel TTN variant, as well as respective phenotypes linked to two other rare variants in the SCN5A and LDLR genes.

## 2 Case presentation

**Figure 1:**
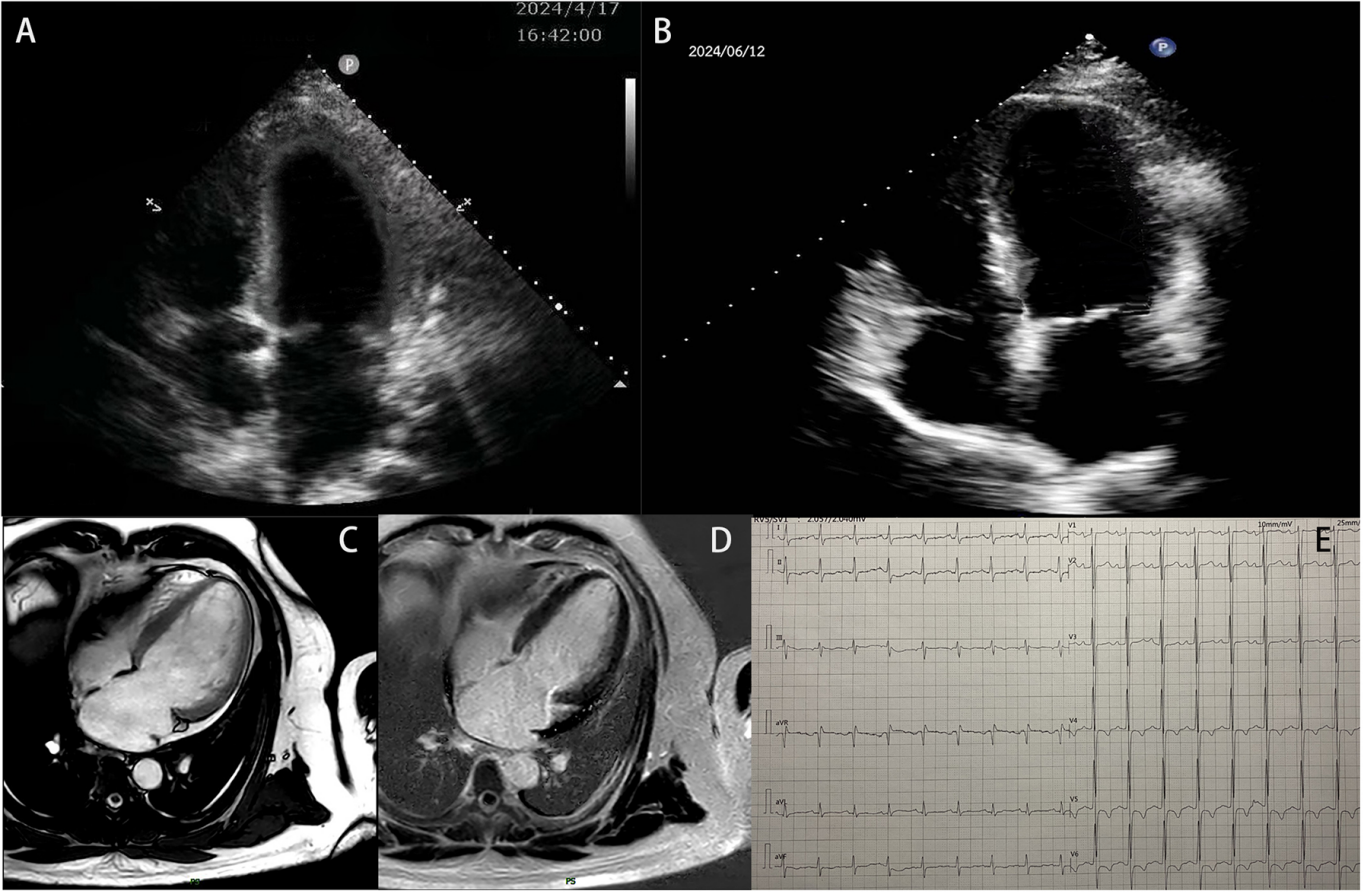
The patient's examination results. **(A)** Echocardiographic results of the patient's heart at hospitalization. **(B)** Echocardiographic results of the patient's heart at the 2-month follow-up. **(C,D)** Images from a cardiac MRI examination. **(E)** Electrocardiogram result.

The proband was a 32-year-old man with a 1-year history of exertional dyspnea who presented to the emergency room with worsening dyspnea and orthopnea over the past 4 days. He had a history of hyperlipidemia and hypertension. He denied smoking, drug or alcohol abuse, and had no family history of cardiac disease or sudden cardiac death at a young age. His blood pressure was 139/84 mmHg and his heart rate was 101 bpm. Electrocardiography (EKG) showed sinus tachycardia, high R waves in precordial leads, and slight ST segment depression with T-wave inversion in leads V4 to V6 (Figure 1E), suggestive of structural heart disease or subendocardial ischemia. Chest X-ray revealed pulmonary edema. Echocardiography revealed left ventricular end-diastolic diameter (LVED) of 61 mm, left atrial diameter (LAD) of 54 mm, right ventricular end-diastolic diameter (RVD) of 46 mm, interventricular septal thickness at diastole (IVsd) of 12 mm, left ventricular posterior wall dimensions (LVPWd) of 12 mm, and left ventricular ejection fraction (LVEF) of 26% (Figure 1A). Laboratory tests showed neutrophilic leukocytosis (WBC 5.87 × 10^9^/L, neutrophils 79.5%, no eosinophilia), hyperlipidemia [cholesterol 529 mg/dl, triglyceride 157 mg/dl, low-density lipoprotein cholesterol (LDL-C) 362 mg/dl], and increased B-type natriuretic peptide (BNP) (1,330 pg/ml). Other tests, including thyroid function tests, iron status, or markers of systemic auto-immune disease, were all normal.

The patient was initially hospitalized and showed improvement after 10 days of treatment with furosemide, spironolactone, bisoprolol, sacubitril/valsartan, dapagliflozin, and recombinant human brain natriuretic peptide. Follow-up echocardiography revealed an LVED of 63 mm, LAD of 51 mm, and LVEF of 44%. To rule out ischemic cardiomyopathy, coronary computed tomography angiography (CTA) was performed, which showed no evidence of obstructive coronary artery disease. A 24-h Holter EKG captured occasional atrial premature contractions. Cardiac magnetic resonance imaging (MRI) revealed an enlarged left ventricle end-diastolic volume (EDV) of 198.21 ml, LVED of 68 mm, and depressed LVEF of 45.5%. Left ventricular wall thickness, T1 mapping values, and extracellular volume were normal. However, multifocal delayed enhancements were observed in the left ventricular septal, lateral, and inferior wall (Figures 1C,D).

**Figure 2:**
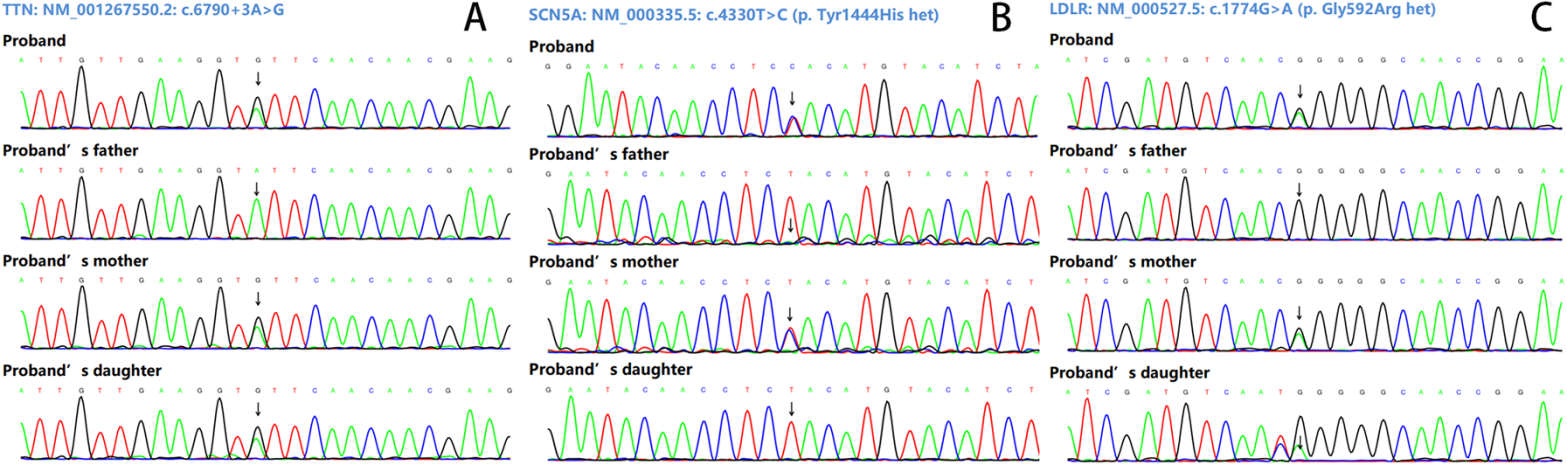
Sanger sequencing results. **(A)** The variant NM_001267550.2: c.6790+3A>G of the TTN gene. **(B)** The variant NM_000335.5: c.4330T>C (p. Tyr1444His het) of the SCN5A gene. **(C)** The variant NM_000527.5: c.1774G>A (p. Gly592Arg het) of the LDLR gene.

**Figure 3:**
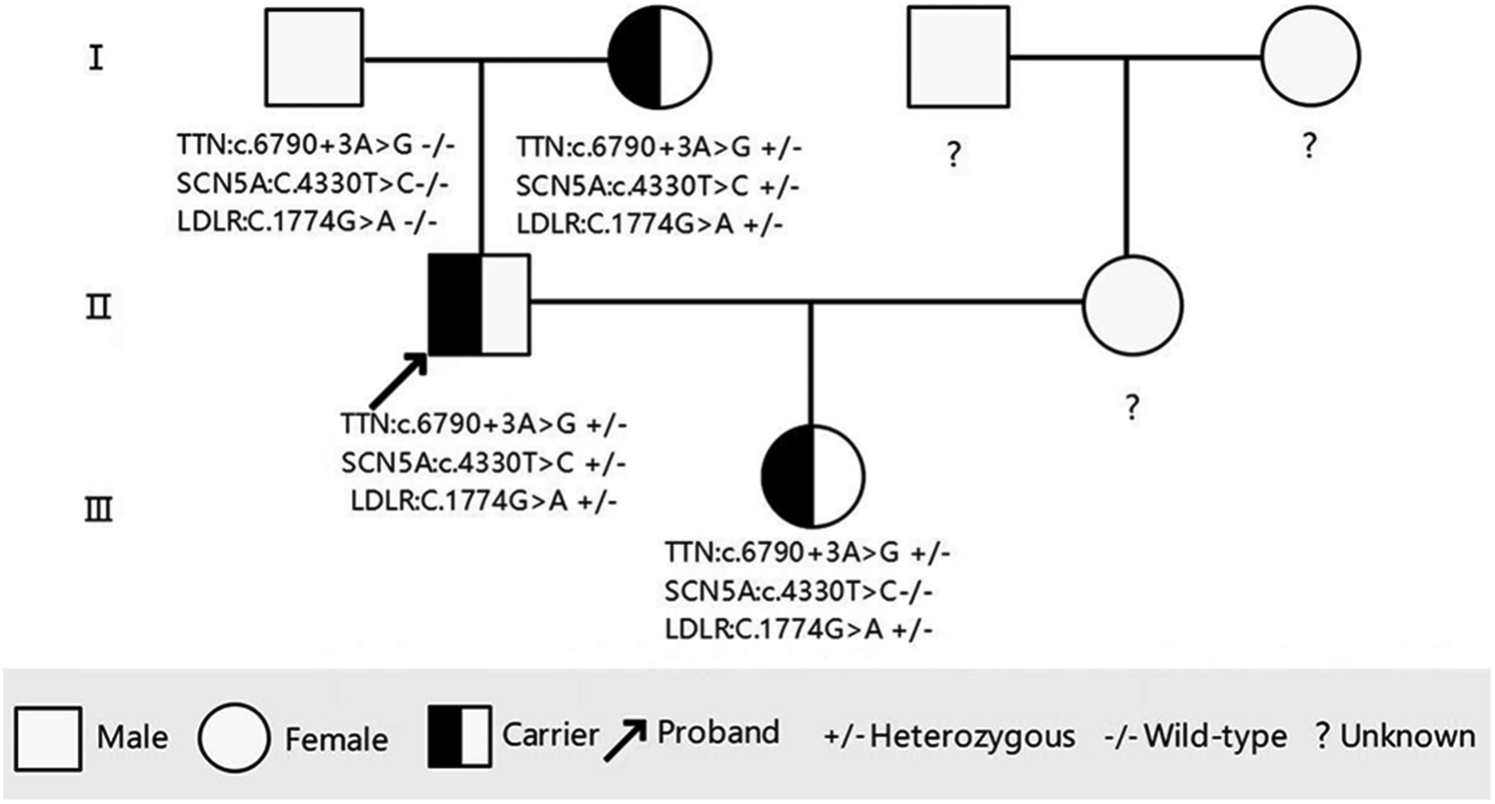
Pedigree of the family showing the genotypes of the proband and affected relatives. Male family members are represented by a square and female family members are represented by a circle.

Based on the imaging results, the proband was diagnosed with DCM. After obtaining ethical approval and written informed consent, target-region capture and second-generation high-throughput sequencing were conducted to analyze all exons and flanking regions. Rare and known variations were validated using Sanger sequencing and cascade screening was performed on all available relatives. The genetic analysis identified a novel heterozygous variant NM_001267550.2: c.6790+3A>G in exon 29 of the TTN gene, a heterozygous variant NM_000335.5: c.4330T>C (p. Tyr1444His het) in exon 25 of the SCN5A gene, and a heterozygous variant NM_000527.5: c.1774G>A (p. Gly592Arg het) in exon 12 of the LDLR gene in the proband. His mother carried all three variants but declined further testing, including echocardiography for DCM detection or blood tests for hyperlipidemia. His daughter carried the TTN c.6790+3A>G and LDLR c.1774G>A variants but was too young at 2 years old to undergo more tests. His father did not carry any of the three mutations (Figures 2, 3).

At discharge, the patient was prescribed furosemide, spironolactone, bisoprolol, dapagliflozin, sacubitril/valsartan, aspirin, and vericiguat. He was followed up regularly at 1 and 2 months, showing stable symptoms, significant relief from shortness of breath, significantly improved activity tolerance, with NYHA class I status. Echocardiography at 2 months showed left ventricular reverse remodeling (LVRR), with a LVED of 60 mm, LAD of 44 mm, IVsd of 11 mm, LVPWd of 11 mm, and LVEF of 50% (Figure 1B).

## 3 Discussion

Titin, encoded by the TTN gene, is the largest human protein. It can be considered a molecular bidirectional spring, contributing to the contraction and relaxation of striated muscles. In addition, titin is involved in sarcomere organization, force transmission and transduction, and signaling responses (4).

Recent landmark sequencing studies of large patient cohorts revealed that TTN mutations are responsible for approximately 20% of all DCM cases (5). Most pathogenic TTN variants are thought to be truncating variants. Titin-truncating variants (TTNtv), including reading frameshifting insertions and deletions, premature termination codons, and splice site mutations, are estimated to account for 15%–25% of inherited DCM cases (6). The penetrance of the DCM phenotype in individuals with TTNtv increases with age, reaching 100% by 70 years. DCM patients with TTNtv variants tend to have worse LV dysfunction and LV dilation compared to those without a genetic diagnosis. Male sex and EF are independent predictors of adverse events (7). In addition, TTNtv is the strongest independent positive predictor of LVRR (8), though LV systolic function may deteriorate in the long term (7). Further, TTN mutations increase the risk of atrial and ventricular tachyarrhythmias (1). A large cohort study found that TTNtv is a significant genetic predisposition factor for alcoholic cardiomyopathy (ACM). The combination of TTNtv and excessive alcohol consumption was associated with worse LVEF in DCM patients (9).

The TTN gene is a classic pathogenic gene associated with DCM. The proband carried a heterozygous variable splicing variant, TTN c.6790+3 A>G, which was absent from the population frequency databases (1,000 Genomes: none, ESP6500: none, ExAC: none). The predicted result using SpliceAl software was 0.85 (range 0–1, >0.2 affects splicing). The dbscSNV-ADA and dbscSNV-RF scores were 0.9989 and 0.89, respectively (scores >0.6 are considered likely to change the shear). These results suggest that this variant may affect splicing. Moreover, this variant has not been reported in either the ClinVar or HGMD databases.

According to the American College of Medical Genetics and Genomics (ACMG) guidelines (10), certain variants (e.g., non-sense, frameshift, canonical ±1 or 2 splice sites, initiation codon, and single- or multi-exon deletion) are often assumed to disrupt gene function by leading to the complete absence of the gene product by lack of transcription or non-sense-mediated decay of an altered transcript. The novel TTN splicing variant is near a canonical splice site (+3) and, based on predictions, may affect splicing in two ways: first, by causing a 282 bp deletion in exon 29, resulting in exon skipping, or second, by causing a 159 bp deletion, resulting in a partial loss of exon 29.

Based on existing evidence, this variant is rare and multiple lines of computational evidence predict it may affect splicing. Since loss of function is the main pathogenic mechanism, this variant may lead to loss of protein function. Although it is classified as a variant of uncertain significance under ACMG guidelines (10), it meets three criteria suggesting possible pathogenicity: PM1 (located in a mutational hot spot), PM2 (absent from controls), and PP3 (multiple lines of computational evidence support the variant influence splicing). However, it lacks familial linkage and functional evidence to support this.

The SCN5A gene encodes the pore-forming ion-conducting α-subunit of the cardiac sodium channel (Nav1.5), responsible for the initiation and propagation of action potentials, and thereby determines cardiac excitability and conduction of electrical stimuli through the heart (3). Diseases associated with SCN5A mutations include the long QT syndrome (LQTS), Brugada syndrome (BrS), isolated (progressive) conduction defect (Lev–Lenègre syndrome), atrial fibrillation, sick sinus syndrome, multifocal ectopic premature Purkinje-related complexes, and DCM. Approximately 2% of patients with DCM cases are caused by SCN5A gene mutations (11). Most SCN5A-linked DCM cases exhibit severe conduction defects and left or right bundle branch blocks. Moreover, arrhythmias are described in over 90% of cases, mainly involving atrial fibrillation, sick sinus syndrome, premature ventricular complexes, and sometimes ventricular tachycardias (12). The presence of atrioventricular conduction defects and supraventricular or ventricular arrhythmias may suggest SCN5A involvement in the DCM diagnosis (13). When an SCN5A mutation is identified, amiodarone may be considered for managing supraventricular or ventricular arrhythmias (14).

The proband carried a c.4330T>C heterozygous missense variant in the SCN5A gene (SCN5A: p. Tyr1444His het), which is extremely rare in populations according to frequency databases (1,000 Genomes: none, ESP6500: none, ExAC: 6.331 × 10^−5^). We used biological information prediction software (including SIFT and Polyphen-2, etc.) and the results all indicated potential harm (SIFT = “D,” Polyph En-2 = “D,” Mutation Taster_pred = “D,” VEST4 score = “0.932,” REVEL score = “0.987,” others = 7 D/1 H), indicating that this amino acid change may impact protein function. The variant substitutes the polar uncharged tyrosine with a polar positively charged histidine. The amino acid at this position is highly conserved among vertebrates. Upon consulting the ClinVar database, the variant was classified as of “Uncertain significance,”, while the HGMD database did not list this variant. Several variants near this locus, including c.4309C>T (p.Pro1437Ser) in Brugada syndrome type 1 and c.4336T>C (p.Tyr1446His) with no associated disease, were classified as suspected pathogenic variants. Given the conservation of this amino acid in vertebrates, it could be a suspected pathogen, but further evidence is needed.

Since TTN is the most common disease gene associated with DCM (accounting for up to 25% of cases), and the proband’s phenotype is consistent with TTNtv-linked DCM (LVRR) rather than SCN5A-linked DCM (no severe conduction defects or left or right bundle branch block), the TTNtv c.6790+3A>G variant is likely the pathogenic variant in this case. However, given that occasional atrial premature contractions were captured in the 24-h Holter EKG, routine ambulatory heart rhythm monitoring is advised to monitor for potential SCN5A-linked arrhythmias.

A previous study examined a family of four generations with autosomal dominant familial DCM and identified an SCN5A variant (c.1003T>C; p.C335R) in all affected family members with either DCM or conduction disease. In addition, a novel truncating TTN variant (p. Ser24998LysfsTer28) could also be identified in two family members with DCM. One patient in particular, with both SCN5A and TTNtv, showed a more severe phenotype. This case demonstrates that the co-existence of multiple genetic variants can affect the severity of the phenotype (15).

FH is a common genetic disorder that increases the risk of premature atherosclerotic cardiovascular disease due to lifelong exposure to high levels of LDL-C. FH is the leading genetic cause of premature cardiovascular disease (CAD). Pathogenic and likely pathogenic variants in the LDLR gene include non-sense, missense, and some synonymous variants, as well as variants in the promoter and canonical splice sequences, small insertions and deletions, and large DNA rearrangements (16). The specific type of pathogenic variant and its severity are linked to the degree of hypercholesterolemia and the risk of CAD development, including premature CAD risk. LDLR null variants are the most severe and non-null variants generally have a milder phenotype (17, 18). Homozygotes display more severe phenotypes than heterozygotes, with plasma cholesterol concentrations typically two to three times higher than normal in heterozygotes (350–550 mg/L) and six to eight times in homozygotes (650–1,000 mg/L). Heterozygotes often develop coronary artery disease after the age of 30 years, while homozygotes often present in childhood.

The proband carried a heterozygous missense variant in the LDLR gene, c.1774G>A (LDLR: p.Gly592Arg het), which is extremely rare in populations according to the frequency database (1000 Genomes: none, ESP6500: none, ExAC: 2.471 × 10^−5^). We again used biological information prediction software (including SIFT and Polyphen-2), all of which were found to be harmful (SIFT = “D,” Polyphen-2 = “D,” MutationTaster_pred = “D,” VEST4 score = “0.930,” REVEL score = “0.991,” and others = “7 D/1 H”), suggesting that this mutation may cause the amino acid change. The amino acid change from non-polar glycine to polar positively charged arginine may impact protein function. Upon querying the database, it was found that the amino acid at this location is also well conserved in vertebrates. Querying the ClinVar database revealed that the variant was classified as “Likely pathogenic” by the reporter, while the HGMD database classified it as a “Disease-causing mutation.” Missense mutations near this locus, such as 1738T>C (p. Ser580Pro), c.1739C>T (p. Ser580Phe), 1747C>G (p. His583Asp), and 1748A>G (p. His583Arg), were identified as pathogenic or suspected pathogenic mutations of FH type 1 by multiple reporters (ClinVar database).

Since the LDLR gene c.1774G>A variant is classified as likely pathogenic, the proband should undergo close clinical follow-up with regular lipid related examinations and, if necessary, coronary CT scans. Diet modification, intensive lipid-lowering treatment, smoking cessation, weight control, and physical exercise are all required.

Given that DCM patients with TTN variants experience worse LV dysfunction and dilation, and the co-existence of multiple genetic variants may worsen the phenotype, the proband may face a poor prognosis. He may need non-pharmacological treatments in the future, such as an implantable defibrillator, a ventricular assist device, or even a heart transplant. He remains under close follow-up.

## 4 Conclusion

In summary, we reported a rare case of dilated cardiomyopathy with three rare genetic variations, including a novel mutation in the TTN gene. These findings enrich the mutation spectrum of TTN both in China and globally. Prenatal advice was offered to the family for any future pregnancies.

The limitations of this study include the lack of validation for expression levels and functional research, as well as the limited number of cases analyzed.

## Data Availability

The original contributions presented in the study are included in the article/Supplementary Material, further inquiries can be directed to the corresponding authors.
